# Nurse practitioner interactions in acute and long-term care: an exploration of the role of knotworking in supporting interprofessional collaboration

**DOI:** 10.1186/s12912-015-0102-x

**Published:** 2015-10-14

**Authors:** Christina Hurlock-Chorostecki, Mary van Soeren, Kathleen MacMillan, Souraya Sidani, Faith Donald, Scott Reeves

**Affiliations:** School of Nursing, Western University, London, ON Canada; School of Nursing, Dalhousie University, Halifax, NS Canada; School of Nursing, Ryerson University, Toronto, ON Canada; Health, Social Care & Education, Kingston University & St George’s, University of London, London, UK

## Abstract

**Background:**

Interprofessional care ensures high quality healthcare. Effective interprofessional collaboration is required to enable interprofessional care, although within the acute care  hospital setting interprofessional collaboration is considered suboptimal. The integration of nurse practitioner roles into the acute and long-term care settings is influencing enhanced care. What remains unknown is how the nurse practitioner role enacts interprofessional collaboration or enables interprofessional care to promote high quality care.

The study aim was to understand how nurse practitioners employed in acute and long-term care settings enable interprofessional collaboration and care.

**Method:**

Nurse practitioner interactions with other healthcare professionals were observed throughout the work day. These interactions were explored within the context of “knotworking” to create an understanding of their social practices and processes supporting interprofessional collaboration. Healthcare professionals who worked with nurse practitioners were invited to share their perceptions of valued role attributes and impacts.

**Results:**

Twenty-four nurse practitioners employed at six hospitals participated. 384 hours of observation provided 1,284 observed interactions for analysis. Two types of observed interactions are comparable to knotworking. Rapid interactions resemble the traditional knotworking described in earlier studies, while brief interactions are a new form of knotworking with enhanced qualities that more consistently result in interprofessional care. Nurse practitioners were the most common initiators of brief interactions.

**Conclusions:**

Brief interactions reveal new qualities of knotworking with more consistent interprofessional care results. A general process used by nurse practitioners, where they practice a combination of both traditional (rapid) knotworking and brief knotworking to enable interprofessional care within acute and long-term care settings, is revealed.

## Background

Interprofessional (IP) care, recognized as one important aspect of high quality safe health care [1, 2], requires effective IP collaboration to achieve its delivery [3]. IP collaboration is active and ongoing partnerships of different healthcare professionals (HCP) who work together in solving problems and providing healthcare services [4]. Concepts indicative of IP care include open communication with equal contribution, shared decision-making, collective problem-solving, coordination to enable interdependent work, and developing respectful professional relationships [5]. While nurse practitioners (NP) are increasingly employed in acute and long-term care settings [6–9], a clear understanding of how they enact IP collaboration or enable IP care is currently limited. In an environment where effectiveness and efficiency are valued, it is imperative that there are clear indications for the integration of innovative health human resources, such as NP roles in hospital and long-term care settings.

Previous research employed the concept of “knotworking” to understand the complex, rapid, and task-oriented collaboration within hospital settings [10, 11]. Based in activity theory, the notion of knotworking, characterized as the tying, untying, and retying of separate threads of activity by loosely connected people, is rapid and partially improvised collaboration [12, 13]. In knotworking the knot, or centre of the activity, is quickly constructed and dissolved ‘on the spot’. Engeström states the knot is “unstable” and the locus of initiative of the knot is always changing. Dimensions of knotworking, the repeated situations of short duration, constantly changing participants, rapid accomplishment of intersubjective understanding, distributed control, and coordinated actions [12] are commonly seen in healthcare situations. For example, researchers on a busy medical unit observed terse, transient, and fragmented interactions that resonate with Engeström’s knotworking concepts. [10] Knotworking is a reasonable theoretical approach to understanding IP collaborations necessary in urgent and rapidly changing situations, such as those commonly seen in the fast paced hospital environment. However, studies of knotworking in hospital settings have not included the NP role. Since collaboration within complex healthcare organizations is often perceived as suboptimal [14], it is important to understand how NPs employed in these settings are interacting to enable IP care. To uncover and create this understanding, we aimed to observe everyday interactions of hospital employed NPs with healthcare professionals to answer the following questions. 1) How do NPs engage in interactions to promote IP care, and 2) How do these interactions compare to knotworking.

## Methods

### Study design

An ethnographic approach to data collection was employed, consisting of observations and interviews [15]. Observations of NPs interacting with their colleagues from other professions during their workday were gathered to provide a contextualized view of NP activities related to their social practices and processes influencing IP care. In this paper we primarily report findings from ethnographic observations of NP working practices.

### Sample and setting

Recruitment of hospital employed NPs was undertaken through presentations about the study at local and provincial NP meetings, posting study information on institutional noticeboards and on a NP website. Emails containing study information were also sent to possible participants by their local NP lead. Participants were included in the study if they were: 1) employed as an NP, 2) worked full time, 3) were engaged in providing patient care, 4) had been in the NP role for at least one year, and 5) were registered with NP credentials. Those expressing an interest in the study met with a research assistant (RA) who explained the study, answered questions, and attained an informed written consent.

Participant recruitment occurred at six purposefully selected hospitals and long-term care settings employing multiple NPs. The hospitals were selected to represent different geographic regions of Ontario, Canada and provide a balance of community and academic organisations with affiliated long-term care settings (nursing homes). At each site a study lead advertised the opportunity for NPs to participate in the study. Ethical approval was received from the Research Ethics Board of Ryerson University (#2012-359-1).

In total, 24 NPs took part in this study. In terms of their demographic information, most were Masters prepared (*n* = 22) and female (*n* = 23). Half had practiced in their hospital NP role for two to five years, about one quarter had between five to 10 years of NP experience, and the remainder had more than 10 years of experience. Around half of the NPs worked with inpatients (hospitals) or residents (nursing homes). The remainder worked in hospitals either exclusively with out-patients or with a combination of in-patients and out-patients.

### Data collection

Six RAs, all with social science backgrounds, were trained in ethnographic observation techniques in order to undertake the fieldwork. Training for the RAs consisted of standard research education such as privacy and attaining consent. In addition each RA attended a two day workshop on the basics of ethnography, how to conduct ethnographic observations, observational issues, specifics to record for the study, and how to write up field notes. Early field notes were reviewed by two researchers to ensure consistency of data. One RA shadowed the NP through his/her entire day, noting what the NP was doing and saying, who the NP met, and in what context the interaction occurred. The RA recorded short field notes on a standardized chart to facilitate rapid recording of key information. Specific recording included the time and location of the interaction, the nature of interaction (e.g. discussing plan of care), nature of actions performed, professional category of those interacting, and duration of the interaction. If needed, after an interaction, the RA would ask to the NP to explain its nature to help contextualise the action. When the interaction occurred by a telephone call or email, the RA sought clarification of the nature of the conversation.

When the NP entered a patient room the RA waited for patient consent before entering. Patient consent, or non-consent, was recorded by the RA on the interaction record. At the end of the day, the NP was invited to share if the day reflected a typical work day. The RA expanded on details during breaks or at the end of the observation day using the chart entries to trigger recollections. Shadowing days were chosen by the RA to cover a range of different work days, and confirmed by the NP. In total 384 hours of observational data were gathered through this approach.

### Analytical approach

Field notes were transcribed verbatim. Qualitative analysis software, NVivo 10, was used to aid data exploration. Coding included line by line and constant comparison to categorize observations according to the predetermined analytical framework described below. Frequency within and between observations was used to determine common social behavior patterns and processes employed by NPs. Regular coder reflexivity provided transparency of potential bias. A sociologist and two nurse researchers formally reviewed the analysis at two points. They determined the emerging work at 10 initial observations (5 NPs) plausible, and the final interpretation resonates with emergence of IP collaboration or care in everyday NP interactions. The convergence of thoughts by a variety of researchers supports that the interpretation is truthful and credible.

A predetermined analytical framework was used to guide the analysis. Interactions between NPs and professional colleagues were explored to determine how the interaction represented qualities of knotworking (e.g. rapid negotiation, improvisation, or delegation). Data were analysed to identify whether the NP was the initiator, facilitator, or recipient of the interaction as well as the resulting action. Interactions were also coded by duration and purpose and then compared with the resultant action. Finally each observation day was explored and compared for general social behaviour patterns and processes. Through this approach, interactions lasting a minute or less were coded as *rapid*. These interactions align with Engeström’s [12] concept of “knotworking” where there are separate threads of activity, loosely connected people, and an unstable center or knot [12, 13]. Longer interactions of information sharing and inquiry (occurring over two to three minutes), were termed as *brief. Extended* interactions were patient care related and longer than three minutes while *social* interactions were unrelated to patient care.

## Results

The interactions of 24 NPs are reported in this paper, consisting of 1,284 observed interactions across six hospitals and affiliated long-term care settings. Four types of interactions emerged from the data: brief (*n* = 719); rapid (*n* = 369); extended (*n* = 124) and social (*n* = 72) interactions. For this paper we focus on the two most common forms of interaction.

### Brief interactions

Brief interactions were observed most frequently across the six study sites. As noted above, these included short interactions of one or two way communications lasting *two to three minutes* with the purpose of educating, information sharing, inquiring, or shared decision-making. More than half of the interactions observed were brief. Brief interactions primarily consisted of information sharing and inquiry. Brief shared decision-making, observed less frequently (*n* = 50), occurred predominantly in academic hospitals and long-term care. The following extract provides an example of a brief interaction:

The NP and a nurse are discussing a patient. The NP asks if the abnormal heart rhythm she saw in the patient chart was transient. The nurse confirms that it was. The NP then says she will order [medication name]. She asks how the patient’s blood pressure has been. The nurse replies “normal” and gives a couple of the most recent values. The nurse then brings a chart over for them to review (*approximately 3 min*). (NP 16, site 5).

The NP initiated brief interactions more often than other professionals (*n* = 388). Brief interactions initiated by NPs commonly resulted in interdependent activity whereas brief interactions initiated by other professions seldom had this result. An interdependent activity is more than simple coordination. Activities are interdependent when one professional relies on another to complete an action so their subsequent action will be possible or successful. The following excerpt illustrates interdependent activities of the NP and physiotherapist where the NP action of changing the patient’s dialysis time is required so the patient will be in the best condition for the physiotherapist’s assessment to enable a successful transfer:

The NP begins a discussion with the physiotherapist about a patient who is ready to transfer from their unit. The NP asks the physiotherapist to complete an application for the patient. They then discuss the patient’s ability to participate in physiotherapy. The physiotherapist asks the NP if it is possible to change the patient’s dialysis time to better accommodate physiotherapy “because he is so tired after dialysis he can’t do physio.” The NP says “OK” and proceeds to call the dialysis unit to make the request. (*approximately 3 min*). (NP 20, site 5).

Brief interactions initiated by the NP resulted in shared decision-making and interdependent activities about twice as often as the same type of interaction initiated by other professionals. An additional resultant activity was identified and coded as ‘collective’. A collective action was defined as two or more professionals working together to complete the activity. The remainder of the brief interactions resulted in equal numbers of collective or independent actions irrespective of who initiated the interaction:

A behavioural specialist stops the NP to speak about a patient. They enter a private office. The two take turns discussing the issues and barriers with the patient’s behaviour providing insights and potential solutions. They decide a family meeting is necessary to allow a decision of the course of action. The NP states she will speak with social work and have the meeting arranged. (*approximately 2 to 3 min*). (NP 13, site 3).

During brief interactions the NPs were observed using open body language such as directly facing others and making eye-contact throughout the interactions. NPs were often observed as sharing equally in talking and listening time, and generally used a calm tone. Towards the end of the work day there was a common observation, among most of the NPs studied, of them tiring. Evidence of tiring was identified with behaviours where the NP took longer to make eye contact, sighed audibly, or appeared less willing to carry on a conversation. Although, the RA noted in these instances that the NP’s tone of voice remained calm.

### Rapid interactions

Almost one-third of the interactions observed across the six sites were coded as rapid negotiation, rapid improvisation, or rapid delegation. These were very brief, one or two way interactions, *of a minute or less*, that were transient. NPs initiated 59 % of all rapid interactions. An example of an observed rapid interaction is as follows:

A female nurse enters the room and tells the NP that a patient is doing badly. The NP goes to the chart on the computer and tells her what drug she can give him and when. The NP writes down some notes and the nurse nods and leaves (*approximately 20 s*). (NP 4, site 2).

Almost half of the rapid interactions were negotiations (*n* = 164) while delegation was least observed (*n* = 74). NPs initiated rapid delegation far more often than other professionals although they initiated this rapid interaction type less than ten percent of the time. Unlike brief interactions, NPs and other professions initiated rapid negotiations with equal frequency. NPs initiated improvisations as commonly as they initiated rapid negotiations, whereas other professions initiated improvisation less. The following rapid negotiation excerpt was observed:

The NP enters the gym on the patient unit and immediately approaches the physiotherapist. In a casual tone of voice the NP states “Hey [physiotherapist’s name], we need to set up a family meeting for [patient name]. When are you free Tuesday or Wednesday next week? The physiotherapist looks up his schedule and gives the NP times he is available. The NP ends the conversation with a smile and “thanks buddy” before walking to the other side of the gym and approaching one of the occupational therapists to ask the same question. (*each interaction was approximately 15 s*). (NP 9, site 3).

Rapid improvisation initiated by the NP most commonly resulted in independent actions. Rapid delegation initiated by the NP almost exclusively resulted in independent actions. The use of rapid delegation was more commonly observed in academic hospitals. NP initiated rapid negotiations were used across all settings equally and often resulted in interdependent actions as illustrated in the following excerpt:

The NP, social worker and occupational therapist are together in a room. The NP has been reviewing a patient chart. The NP says “if [patient] isn’t compliant with going to the family doctor, I can do a little physical assessment and [occupational therapist’s name] will assess for risk of falls.” They all nod in agreement and take notes. (*approximately 1 min*). (NP 17, site 5).

The NP participants were observed to use four types of interactions throughout their day. A generalized process of transitioning from interactions aimed to gather information to interactions that aim to provide information emerged. For the NPs working with in-patients the transition occurred over the entire day while those who worked in an ambulatory setting the transitioning process occurred with each patient appointment. This transitioning behaviour pattern, observed across all six sites, provides an understanding of how NPs use rapid and brief interactions to enable IP collaboration and care.

### Extended and social interactions

As noted above, in contrast to rapid and brief interactions, extended and social interactions were only occasionally observed across the six hospitals. Extended interactions consisted of walk-around rounds where two or more professions walked from patient to patient discussing care, team rounds where several professions sat in a room to discuss patient care, family meetings, or formal education sessions. Social interactions were short pleasantries or brief interactions not related to patient care or hospital business. The few observations did not augment understanding of the NP process of enabling IP collaboration or care.

## Discussion

Through the collection of observational data at six sites, this study reveals how NPs in hospital and long-term care settings augment IP collaboration through traditional knotworking (rapid interactions), and a new form of knotworking (brief interactions). Brief interactions reveal new qualities of knotworking and a consistent result of IP care elements. These findings suggest there may be different forms of knotworking rather than the single version as described by Engeström [12]. How NPs use these forms of knotworking is also revealed as new understanding of the NP role and provides new insight into the role’s value in acute and long-term care settings.

Knotworking, in its original form is characterized as the tying, un-tying, and re-tying of separate threads of activity by loosely connected people [12]. Rapid knotworking techniques observed in the current study primarily resulted in independent care activities much like the terse, transient, and fragmented interactions described by Reeves and Lewin [10]. The rapid knotworking observed seldom involved multiple people, therefore resulted in a fragmented or siloed approach to care, rather than IP care. Yet, rapid knotworking is necessary in urgent and rapidly changing situations, such as those commonly seen in the fast paced hospital environment. For example, a life threatening situation where a patient has a sudden change in his/her ability to breathe requires rapid delegation and perhaps rapid improvisation for a successful outcome. However, the continuous use of rapid knotworking raises concerns. When the ‘knot’ is rapidly formed and dissolved, it allows the creation of gaps in communication among the many different professions involved with the patient. As a result, duplication occurs and care decisions and delivery become fragmented over time. NPs in this study were observed initiating all types of rapid knotworking although the rapid negotiations they initiated had a different result. NP initiated rapid negotiations usually resulted in interdependent activities. This suggests NPs use a different approach with rapid negotiation that enables IP collaboration and promotes IP care. The HCPs who work with NPs described two key attributes of NP roles that may explain how IP collaboration is enabled. First is the consistency of the NP role within a team of changing professionals where the NP holds a more comprehensive knowledge of the patient. The HCPs describe this consistency as positioning the NP in the centre as a hub allowing HCPs access to patient knowledge that is clear and consistent. Second is the ability of the NP role to link or bridge across professions. For rapid negotiation, the most valued bridging is the legal authority of the NP role to make medical decisions thus enabling timely care changes. This is consistent with recent research where the extended NP knowledge and authority was found to influence timely and safe patient care [16, 17]. The use of rapid knotworking in hospital and long-term care settings is not new, although how the NPs use rapid negotiation to enable IP activity is. Further to knotworking is the more frequently used brief interaction technique with characteristics and dimensions that differ from the traditional understanding of knotworking.

The observation of brief interactions used by the NPs in this study offers a new insight into the utility of these interactions as a second form of Engeström’s knotworking. We will refer to these interactions as “brief knotworking”. Brief knotworking retains some of the rapid knotworking characteristics such as repeated situations, constantly changing participants, distributed control, and coordinated actions. Yet brief knotworking has the additional qualities of longer interaction duration, a more consistent locus of initiation, and retention of the essence of the interaction. In the current study, the locus of initiation of brief knotworking was most frequently associated with the NP role. The NP initiated brief knotworking with multiple HCPs throughout their day. In brief knotworking the knot, or centre, is not completely dissolved at the end of the interaction. Instead, in the un-tying process, the NP retains a segment of the thread as the ‘essence of the knot’. In retaining the essence of the knot, the NP stabilizes the knot and retains the locus of initiation of further related knots. The tying, un-tying, and re-tying continue to occur although the NP is now twining the retained threads to create a strong ‘IP cord’. This IP cord is used in subsequent brief knotworking. These additional characteristics of brief knotworking (longer interaction duration, consistent locus of initiation, and retention of the essence of the knot) increase the ability to promote IP collaboration and care.

**Fig. 1:**
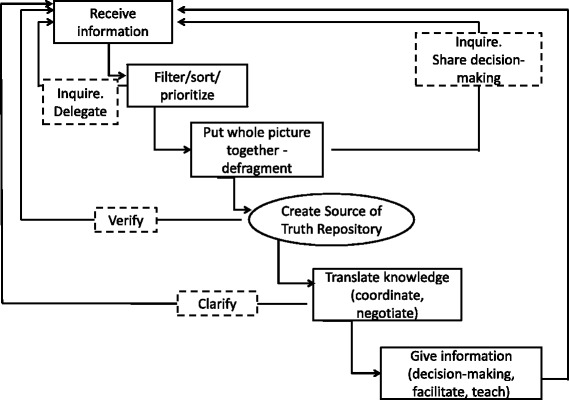
The NP interprofessional knotworking process. This figure captures a simplistic view of the IP knotworking process used by NP. While the figure appears stepwise, the process is iterative. The process begins with receiving or making connections (tying knots) to gather information. The NP retains a thread from each brief knotworking interaction essentially filtering, sorting, and prioritizing each thread. Inquiry is used to aid in the filtering. When required, delegation of a task may occur at this point. The retained threads of information are twined together. To ensure a strong IP cord of information the NP may engage in further inquiry or initiate share decision-making with other HCPs. In doing this, the NP defragments the information and creates a comprehensive, holistic picture of patient needs and responses from the healthcare professional perspectives. The retained IP cord eventually becomes a collection of validated knowledge termed the “source of truth repository” and a collective IP intentionality of the plan of care is created. Next the NP uses brief knotworking to deliver the knowledge back to all involved. In re-tying the strong IP cord of information back to other professionals, the NP translates knowledge, aids in coordination, and provides a clear, IP understanding. The process requires the use of both rapid and brief knotworking to encourage partnerships, collective problem-solving, clear and timely communication, shared decision-making, and coordination for interdependent activity. In the outpatient setting the NP completed the process with each patient’s visit. In the inpatient setting the NP juggled several processes consecutively with each process at a different stage

Many HCPs engaged in, or initiated, brief knotworking but not all brief knotworking interactions resulted in IP care. Exploration of the NP day for general social patterns revealed a process of employing brief and rapid knotworking that may better explain how they enable IP care (see Fig. 1). The process begins with the NP receiving information or making connections (tying knots) to gather information. Rapid knotworking techniques provide aspects of information in certain situations although brief knotworking techniques are the main source of inquiry. The NP retains a thread from each brief knotworking interaction essentially filtering, sorting, and prioritizing each thread. The retained threads are twined together creating a stronger IP cord of information for re-tying in subsequent interactions. In doing this, the NP defragments the information and creates a comprehensive, holistic picture of patient needs and responses from the healthcare professional perspectives. With a holistic IP understanding, the NP continues with knotworking techniques of inquiry and shared decision-making. The retained IP cord eventually becomes a collection of validated knowledge we termed the “source of truth repository” and a collective IP intentionality of the plan of care is created. While this source of truth is important, the NPs were found to continue using brief knotworking to deliver the knowledge back to all involved. In re-tying the strong IP cord of information back to other professionals, the NP translates knowledge, aids in coordination, and provides a clear, IP understanding. The process requires the use of both rapid and brief knotworking to encourage partnerships, collective problem-solving, clear and timely communication, shared decision-making, and coordination for interdependent activity.

The HCPs expressed views of the NP role that suggest two conditions exist when NP knotworking results in IP collaboration and care. The first condition is the type of IP relationship that the HCPs experience. Relationships where the NP role acted as a link between professions, translating information and knowledge as an equal, were associated with smoother group functioning and seamless care. However, when the NP employed a hierarchical position above other HCPs or when the physician held a strict dominant position above the NP, there was less shared decision-making and more delegation. Social interactions can be used to develop the professional relationships needed to enable IP collaboration. The observed social interactions initiated by NPs are likely the means of creating the personal and professional trust needed to maintain a positive role image. The development of trust has been clearly identified as necessary for successful IP relationships [16, 18, 19].

The second condition is quality communication. The HCPs expressed two highly valued NP attributes that they perceived enabled IP care. Availability was the most valued attribute described by HCPs. When the NP was easily accessible for questions and medical care decisions, HCPs felt patient care was seamless and timely. Being available allows the NP to engage in communication and coordination to enable interdependent activities thus group functioning was perceived as smoother and workday stress reduced. Accessibility has been identified in recent research as key to building smooth functioning groups [20]. The second attribute, the consistent NP presence within a group of constantly changing members, influenced greater communication and was perceived to enhance collaboration and cohesion amongst the group. Consistency of the role within the group positions the NP to accept the multiple threads of information and retain the essence of the knot. Quality outcomes linked with NP role consistency are evident in recent research [21–23].

The observations of fragmented knotworking [10] and suboptimal interprofessional collaboration [14] in earlier research may be apparent because there was not a role, such as the NP, integrated into these teams. In the current study the NP role was found to be balancing two types of knotworking to enable IP care. The use of both types of knotworking allows for richer connections, clearer communication, and results in high quality care in hospital and long-term care settings. There are, inevitably, limitations to this study. The study was limited to observing and examining only those interactions engaged in by the NP participants. The quality of communication during interactions was not assessed. Not all the NP’s interactions could be observed, such as interactions within the patient room if the patient did not consent to observation. This means a number of exchanges may have been missed. This research took place within one Canadian province and therefore generalizability of the findings is cautioned.

## Conclusion

This study reveals a new insight into the notion of knotworking and how it is used to enhance IP care within acute and long-term care settings. As outlined above, there are two forms of knotworking initiated by NPs in their IP work: *rapid* and *brief*. Rapid knotworking is valuable since it rapidly addresses and solves problems important in healthcare environments where rapidly changing patient conditions are prevalent. Brief knotworking is more coordinated and integrated and more consistently results in IP collaboration and care. NPs employed both types of knotworking within a general process to solve problems, enable timely care provision, reduce duplication, decrease errors caused by fragmented communication, and move the collective intentionality of the plan of care consistently forward. These differing types of knotworking need to be explored further to more fully understand their uses and impacts.

## Abbreviations

IP: Interprofessional
HCP: Healthcare professional
NP: Nurse practitioner
RA: Research assistant
